# Fabrication, characterization and optical properties of Au-decorated Bi_2_Se_3_ nanoplatelets

**DOI:** 10.1038/s41598-022-22408-5

**Published:** 2022-10-22

**Authors:** Chih-Chiang Wang, Yu-Sung Chang, Pao-Tai Lin, Fuh-Sheng Shieu, Han-Chang Shih

**Affiliations:** 1 Department of Materials Science and Engineering, National Chung Hsing University, Taichung, 40227 Taiwan; 2 Department of Electrical and Computer Engineering, Texas A&M University, College Station, TX 77843 USA; 3 Department of Chemical Engineering and Materials Science, Chinese Culture University, Taipei, 11114 Taiwan

**Keywords:** Materials science, Nanoscience and technology

## Abstract

Au-decorated Bi_2_Se_3_ nanoplatelet heterostructures are fabricated by a two-step process of thermal CVD at 600 °C and magnetron sputtering at room-temperature. The crystal structures and binding energies of rhombohedral Bi_2_Se_3_ and FCC Au are determined by XRD, HRTEM, XPS, and Raman spectroscopy. XPS and Raman spectroscopy reveal the interaction between Au and Bi_2_Se_3_ by shifting in the binding energies of Au–Au, Au–Se and Bi–Se bonds and the wavenumber of A_1g_^2^ and E_g_^2^ modes. Au-decorated Bi_2_Se_3_ nanoplatelet heterostructures are observed using FESEM, and confirmed by XPS, Raman spectroscopy, and HRTEM imaging. Their optical band gap of the Au-decorated Bi_2_Se_3_ nanoplatelet heterostructures increases with Au thickness about 1.92-fold as much as that of pristine Bi_2_Se_3_ (0.39 eV), owing to the Burstein-Moss effect. The optical absorptance of the Au-decorated Bi_2_Se_3_ nanoplatelet heterostructures revealed increment with wavelength from 200 to 500 nm and decrement with increasing wavelength from 500 to 800 nm.

## Introduction

Heterostructures comprise at least two materials, of which one forms a host material and another is the covering material covered the host material. The materials of the heterostructures can be metals, semiconductors, dielectrics, or polymers^1^. Such a heterostructure can perform the functions of the different materials, including the absorption of light, charge transfer, quantum yielding, and enhancement of the near-field electromagnetic field. Therefore, the potential applications of heterostructures include (1) reduction of the signal-to-noise ratio of analytes^2^, (2) increasing the stability of colloidal particles^3^, (3) increasing the Raman intensity of analytes^4^, (4) detecting biomaterials^5^, (5) transporting drugs^6^, (6) use in high-efficiency photonic crystals^7^, and (7) high-efficiency photocatalysis^8^.

Bismuth selenide (Bi_2_Se_3_) is a direct-band-gap material (~ 0.35 eV) and has a rhombohedral crystal structure^9^. Bi_2_Se_3_ is comprised of layered structure. Each layer in Bi_2_Se_3_ is formed from five stacked atomic layers, Se^1^–Bi–Se^2^–Bi–Se^1^, which are called quintuple layers (QLs)^10^. The covalent force bonds the Se and Bi within each QL; van der Waals´ forces dominate between QLs^11^. Bi_2_Se_3_ is a unique quantum material owing to its gapless surface state and is insulating bulk band gap, both of which are based on time-reversal symmetry and spin-orbital coupling^12,13^. Owing to its unique properties, Bi_2_Se_3_ has many potential applications, such as terahertz detection, visible-IR photodetection, and spin-optoelectronics^14^. The optical band gap of Bi_2_Se_3_ can be tuned by controlling the following factors. (1) The method of synthesis, which can be chemical deposition^15–17^, thermal evaporation^18,19^, or the hydrothermal method^20^, and (2) dopants, such as Te^19,21^, Sb^22^, Pb^23^, Dy^24^, Ni^25^, Sn^11^, Cr^26^, In^11^, Gd^27^, Tl^28^, Fe^29^, and Nd^20^. (3) the heterostructures; these include ZnO@CdS nanostructures^30^, ZnO-ZnS nanostructures^31^, TiO_2_@CdS nanorods^32^, ZnO@Ag nanostructures^33^, silver/ZnO and gold/ZnO nanostructures^34^, and CdSe/ZnS nanocrystals^35^. Therefore, a heterostructure is useful for modifying the optical band gap of Bi_2_Se_3_ nanostructures. The covering material is fabricated from Au, Ag, or Cu. Ag is the most common material for synthesizing the covering material because it has a wider range of optical absorption range than Au and Cu. However, Au is the best material for use in the ambient environment because it is more chemically stable than Ag and Cu^36^. The heterostructures that are based on Bi_2_Se_3_ have been studied; these include Ag@Bi_2_Se_3_ nanoparticles^37^, MnSe@Bi_2_Se_3_ nanoparticles^38^, Au@Bi_2_Se_3_ nanoparticles^39^, Bi_2_Se_3_@mSiO_2_–PEG nanoparticles^40^, CdSe/Bi_2_Se_3_ quantum dots^41^, and ZnSe/Bi_2_Se_3_ core–shell QDs^42^.

In order to understand the effect of the Au in the optical property of the Bi_2_Se_3_ nanoplatelets (NPs), the heterostructures of Au-decorated Bi_2_Se_3_ NPs with various Au thicknesses are fabricated by a two-step process, which involve thermal CVD for synthesizing the Bi_2_Se_3_ NPs and magnetron sputtering for depositing the Au. The optical properties of the band gap and absorptance, crystal structure, and chemical bonding are systematically studied.

## Results

### Surface morphologies

**Figure 1:**
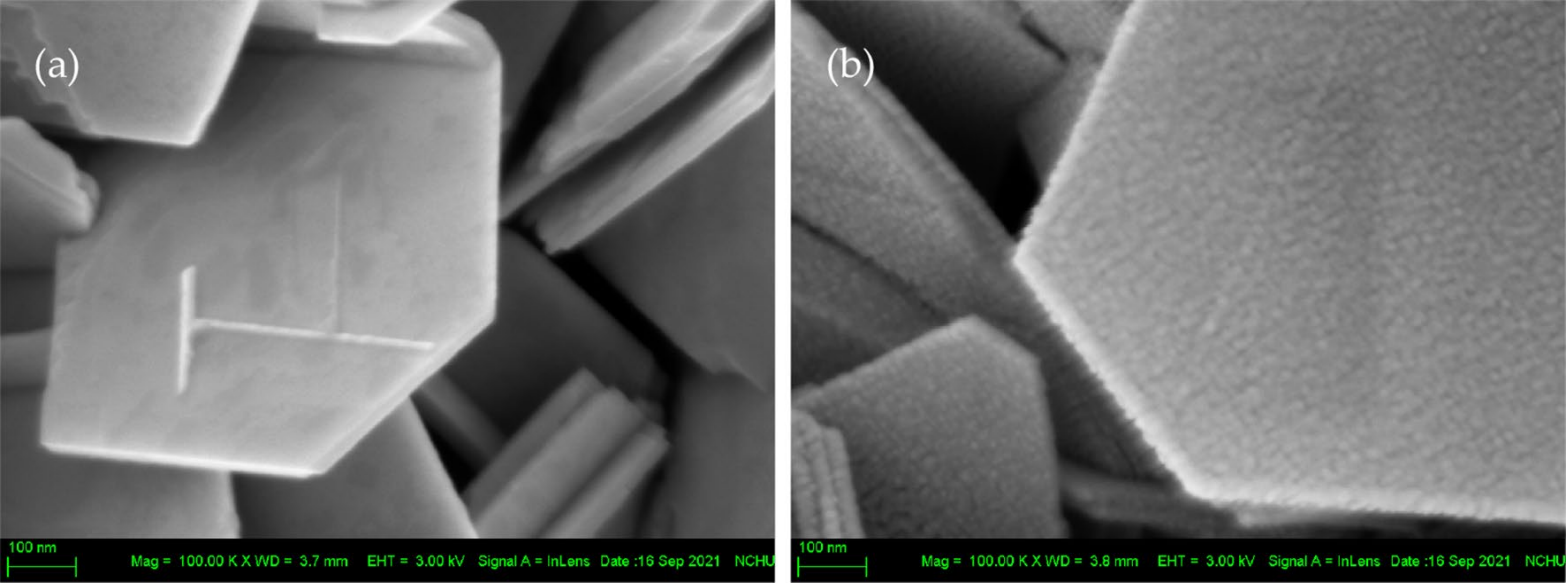
FESEM images of (**a**) pristine and (**b**) Au180s-decorated Bi_2_Se_3_ NPs.

Figure 1a presents the hexagonal-like pristine Bi_2_Se_3_ NPs, which had a flat and smooth morphology. Figure 1b presents the rough surface of the Au180s-decorated Bi_2_Se_3_ NPs. The mean thickness and diameters of pristine Bi_2_Se_3_ and Au-decorated Bi_2_Se_3_ NPs, estimated using Image J software, were 38.38 nm and 55.83 nm, and 698.06 and 816.97 nm, respectively. Table 1 presents the mean thickness and diameters of all of the Au-decorated Bi_2_Se_3_ NPs; these values increase with Au deposition time. Figures S1(a), (b), (c), (d), (e), (f), and (g) present the surface morphologies of all the NPs. The pristine Bi_2_Se_3_ had a smooth surface, while the formed Au-decorated Bi_2_Se_3_ NPs had rough surfaces.

**Table 1:** Thicknesses and diameters of the Au-decorated Bi_2_Se_3_ NPs with various Au deposition time.

Au deposition time (sec)	0	30	60	90	120	150	180
Thickness (nm)	34.38	37.56	39.67	42.94	48.04	54.48	55.83
Diameter (nm)	698.06	712.37	744.35	769.05	796.89	810.56	816.97

### Analysis of crystal structure

**Figure 2:**
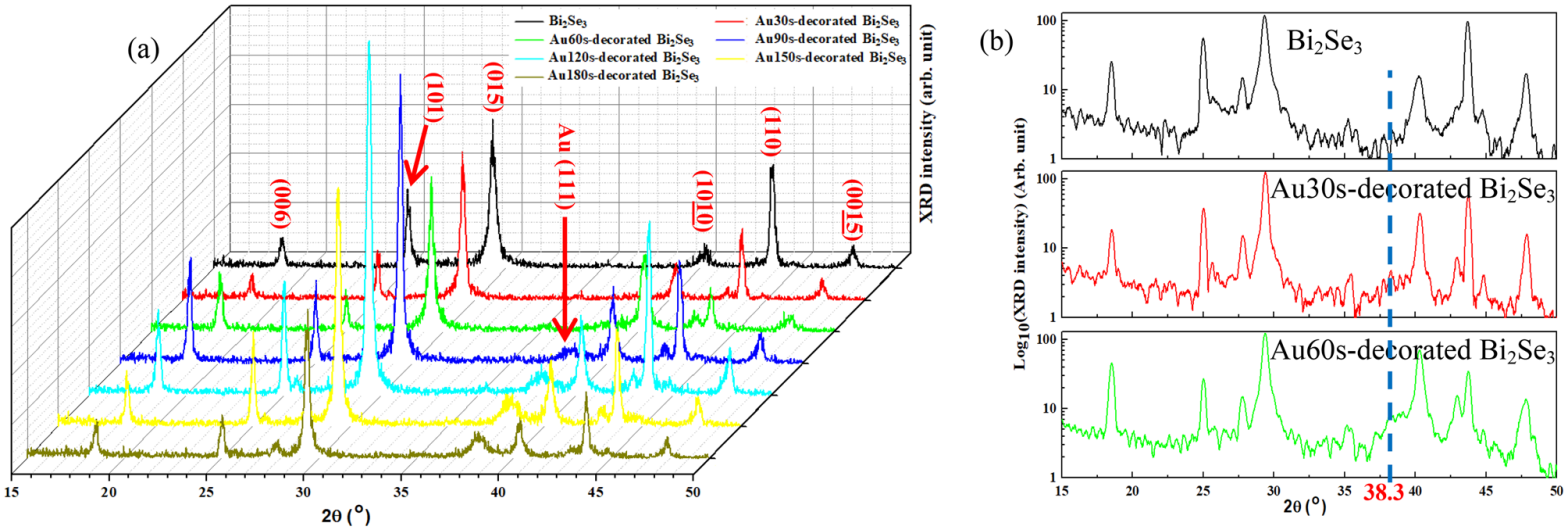
XRD patterns of the pristine Bi_2_Se_3_ and Au-decorated Bi_2_Se_3_ NPs in (**a**) linear and (**b**) log_10_ Y-axis.

Figure 2 presents the XRD patterns of the pristine Bi_2_Se_3_ and Au-decorated Bi_2_Se_3_ NPs, which reveal typical rhombohedral Bi_2_Se_3_ crystal structures (JCPDS 89-2008) with Bi_2_Se_3_ planes (006), (101), (015), (1010), (110), and (0015) at 2θ values of 18.55°, 25.03°, 29.39°, 40.28°, 43.77°, and 47.67°. The Au(111) plane is observed at 38.30° when the Au deposition exceeds 90 s, as shown in Fig. 2a. In Fig. 2b, the Y-axis has a log_10_ scale, and Au30s- and Au60s-decorated Bi_2_Se_3_ NPs yield the Au(111) peak. The lattice constants are estimated as follows.

1 $$\frac{{1}}{{{{d}}_{{\left( {{{hkl}}} \right)}}^{{2}} }}{ = }\left[ {\frac{{4}}{{3}}\left( {{{h}}^{{2}} {{ + k}}^{{2}} {{ + hk}}} \right){{ + l}}^{{2}} \left( {\frac{{{a}}}{{{c}}}} \right)^{{2}} } \right]\frac{{1}}{{{{a}}^{{2}} }}$$

where *h*, *k*, and *l* are the Miller indices, *d*_*(hkl)*_ is the perpendicular distance between adjacent *(hkl)* lattice planes, and a and c are the lattice constants of Bi_2_Se_3_. The grain sizes of the Bi_2_Se_3_ NPs and the Au are estimated using the Scherrer equation. Table 2 presents the grain sizes of the Bi_2_Se_3_(015) and the Au(111), and the lattice constants (*a* = *b*, and *c*) and *c*/*a* ratios of the Bi_2_Se_3_, which are consistent with previously obtained results^11,43,44^. The average grain size of Bi_2_Se_3_(015) was (22.08 ± 14.85%) nm, and the Au grain size increased with the Au deposition time. Au had no effect to change the crystal structure of the Bi_2_Se_3_.

**Table 2:** Lattice constants (a and c) of Bi_2_Se_3_ NPs, and grain sizes of Bi_2_Se_3_ and Au in various Au deposition time.

Au deposition time (sec)	0	30	60	90	120	150	180
a (Å)	4.145	4.132	4.134	4.136	4.135	4.134	4.132
c (Å)	28.745	28.714	28.722	28.671	28.692	28.689	28.709
c/a	6.9434	6.9488	6.9471	6.9315	6.9386	6.9385	6.9468
Bi_2_Se_3_(015) grain size (nm)	18.05	22.74	19.56	24.61	23.62	22.65	23.39
Au(111) grain size (nm)	0	n/a	n/a	4.37	5.91	6.90	7.82

### Fine structure analysis

**Figure 3:**
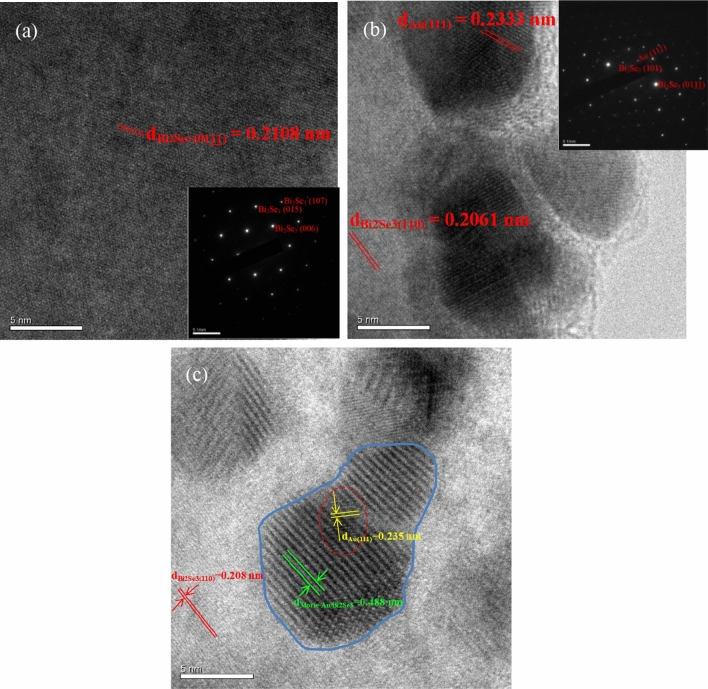
HRTEM images of (**a**) pristine Bi_2_Se_3_, (**b**) Au180s-decorated Bi_2_Se_3_ NPs. Insets are the SAED patterns, and (**c**) the Moiré pattern originating from the superposition of the lattices of Bi_2_Se_3_ and Au.

Figures 3a,b present HRTEM images of the pristine Bi_2_Se_3_ and Au180s-decorated Bi_2_Se_3_ NPs. The d-spacings of 0.2108 nm (Fig. 3a) and 0.2061 nm (Fig. 3b) are corresponding to the planes of Bi_2_Se_3_(0111) and Bi_2_Se_3_(110), respectively. The insets in Fig. 3a,b present the respective HRTEM-SAED (selected-area electron diffraction) patterns. In Fig. 3a, (006), (015) and (107) planes of Bi_2_Se_3_ are observed, and in Fig. 3b, (101) and (0111) planes are observed. The d-spacing of 0.2333 nm in Fig. 3b corresponds to the Au(111) plane. The electron-diffracted spot of Au, seen in Fig. 3b, is observed in the SAED pattern that corresponds to the Au(111) plane. Figure S2 shows low-magnitude TEM images and the Au thickness of the Au-decorated Bi_2_Se_3_ NPs at various Au deposition times. These results confirm that the crystalline Au and Bi_2_Se_3_ were successfully fabricated. Figure 3(c) shows the HRTEM image of the Au180s-decorated Bi_2_Se_3_ NPs. The d-spacings of the Au(111) and the Bi_2_Se_3_(110) planes has been resolved and can be observed in the red circle and outside the blue circle, respectively. The d-spacing inside the blue circle is estimated as the 0.488 nm which is not correspondent to any plane of the Au and Bi_2_Se_3_. This enlarged d-spacing is attributable to the Moiré effect, and forms the Moiré pattern between the lattice plane of Au and Bi_2_Se_3_, which confirms the interaction between the lattice of the Au and Bi_2_Se_3_ crystals.

### XPS analysis

**Figure 4:**
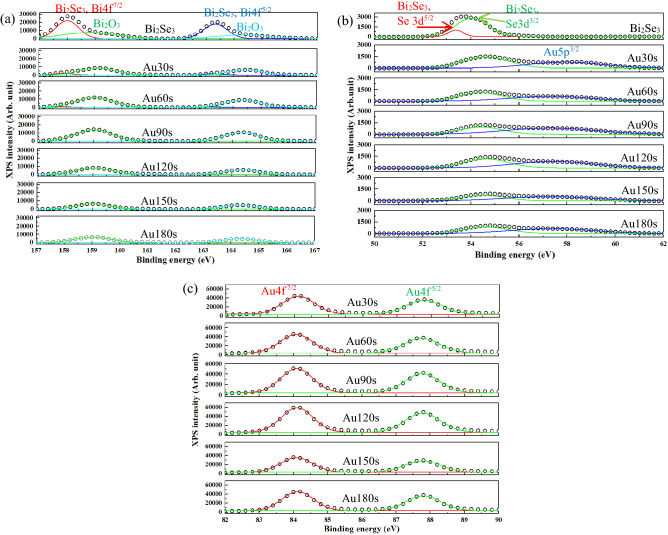
XPS spectra of (**a**) Bi 4f., (**b**) Se 3d, and (**c**) Au 4f. of the Au-decorated Bi_2_Se_3_ NPs.

Figure 4a–c present the XPS binding energies in the orbitals of Bi 4f., Se 3d, and Au 4f., respectively. In Fig. 4a, the peaks at 157.99 eV and 163.31 eV correspond to the Bi 4f^7/2^ and Bi 4f^5/2^ orbitals of the Bi_2_Se_3_ phase^45^, and peaks that belong to the Bi_2_O_3_ phase are observed at 158.79 eV (Bi 4f^7/2^) and 163.31 eV (Bi 4f^5/2^)^46,47^. The intensities of the peaks that correspond to the Bi_2_Se_3_ phase decrease as the Au thickness increase, and the peaks that correspond to the Bi_2_O_3_ phase have similar intensities, as shown in Fig. 4a. XPS reveals the chemical binding energy extremely close to the sample surface (~ 2–5 nm). In this work, the chemical bonding energies on the surface of the samples were directly measured using XPS with Al Kα X-rays (1486.6 eV), without pre-bombardments. The skin depth (*δ*) formula yields the effective depth of penetration into the sample of incident light^48^.

2 $$\delta = \frac{{\lambda_{0} }}{2\pi Im\left( n \right)}$$

where *λ*_0_ is the wavelength in free space. *Im*(*n*) is the imaginary part of the complex refractive index of the metal (Au)^48^.

3 $$Im\left( n \right) = \sqrt {0.5\left( {\sqrt {\varepsilon_{1}^{2} + \varepsilon_{2}^{2} } - \varepsilon_{1} } \right)}$$

where *ε*_1_ is the negative real part of the relative permittivity of the metal, representing the polarization strength; the imaginary part of *ε*_2_ of the relative permittivity represents the metallic loss of the Au metal^48^. The *δ* of the Al kα X-rays is 0.045 nm, which is less than the Au thickness. The XPS intensity is proportional to the photoionization cross-section of the specific level from which the photoionization occurs, a function of the photon energy for any given level, and a function of the path length along which the photoelectrons have to pass in the object to reach the sample surface and be ejected into the vacuum^49^. The XPS intensity of the photoelectron signal (*dI*) is given as^48^

4 $$dI = I_{0} exp\left( { - \frac{x}{\lambda }} \right)dV$$

where *I*_0_ is the average photoionization signal which is constant for the specific electronic level; x is the distance traveled by the photoelectrons within the object before they emerge from its surface; λ (Å) is the mean free path of the photoelectrons during inelastic scattering inside the object, and varies approximately as^49^

5 $$\lambda = \frac{1}{2}\sqrt {KE}$$

*KE* is the kinetic energy of the photoemitted electrons; *dV* is the per unit volume. This is an empirical relation which is valid for the kinetic energy above 150 eV^49^. Based on the above discussion, the photoelectrons traverse an increasing distance inside the samples as the Au thickness increased. The signals that correspond to the Bi_2_Se_3_ phase, therefore become weaker as the Au thickness increases. The Bi_2_O_3_ phase is naturally formed in the ambient environment and covers on the sample surface. The signals that are attributable to the Bi_2_O_3_ phase continue to be observed even as the Au thickness increases. The formation of the Bi_2_O_3_ phase on the Bi_2_Se_3_ surface indicates that the photoelectrons from the Bi_2_O_3_ pass through a shorter distance than those in the Bi_2_Se_3_. The XPS intensities of Bi_2_Se_3_ peaks therefore decreased and those of Bi_2_O_3_ remain observed. Two peaks that correspond to Se 3d^5/2^ and Se 3d^3/2^ of the Bi_2_Se_3_ phase are observed at 53.37 eV and 54.05 eV^45^, as shown in Fig. 4b. The peak at around 57.2–57.9 eV is attributed to Au 5p^3/2^^50,51^. The intensities of Se 3d^5/2^ and Se 3d^3/2^ peaks decrease as the Au thickness increase, as did those of the Bi 3d orbitals owing to an increase in the distance traversed.

Peaks of Au 4f^7/2^ and Au 4f^5/2^ of Au30s, Au60s, Au90s, Au120s, Au150s, and Au180s-decorated Bi_2_Se_3_ NPs are observed at around 84.1 eV and 87.77 eV^52^, as shown in Fig. 4c. The typical binding energy of metallic Au of the orbitals of Au 4f^7/2^ and Au 4f^5/2^ are 83.8 eV and 87.45 eV^53^. Figure 4c shows that the binding energies of Au 4f^7/2^ and Au 4f^5/2^ reveal a slight blue-shift to 84.08–84.13 eV and 87.50–87.79 eV. Nath reported that the orbitals of Au 4f^7/2^ and Au 4f^5/2^ had binding energies of 84.3 eV and 87.9 eV in Au–Se nanoalloys^54^. Hu reported that Au 4f^7/2^ and Au 4f^5/2^ had binding energies of 84.0 eV and 87.6 eV when Se was bonded on the surface of Au nanoparticles^55^. Cueva reported that Au 4f^7/2^ and Au 4f^5/2^ had binding energies of 84.4 eV and 88.0 eV when Au nanoparticles were on the surfaces of CdSe nanopyramids^56^. Mikhlin reported that the binding energy of the Au 4f^7/2^ orbital was blue-shifted from 84.15 eV to 84.7 eV in the cases of Ag_3_AuSe_2_ and Ag_3_AuS_2_ compounds^57^. The above results are attributed to increases in the positive charges of Au atoms. The blue-shift of the binding energy of the Au 4f. orbital is attributable to the following causes. (1) The final state effect, which is characteristic of nanoparticles that have diameters of less than about 5 nm, and (2) the chemical shift that is caused by the transfer of electrons from gold to chalcogen atoms. In this work, Au-decorated Bi_2_Se_3_ NPs have a shell-like structure that is composed of Au nanoparticles with a diameter of 5 nm (Fig. 3b), consistent with the blue-shift of the binding energy of Au 4f. orbitals that has been found elsewhere^52,58^. The blue-shift of the binding energy proves the transfer of electrons and chemical binding between Au and Se. According to the XPS results, the heterostructures of Au-decorated Bi_2_Se_3_ NPs were successfully synthesized.

### Raman analysis

**Figure 5:**
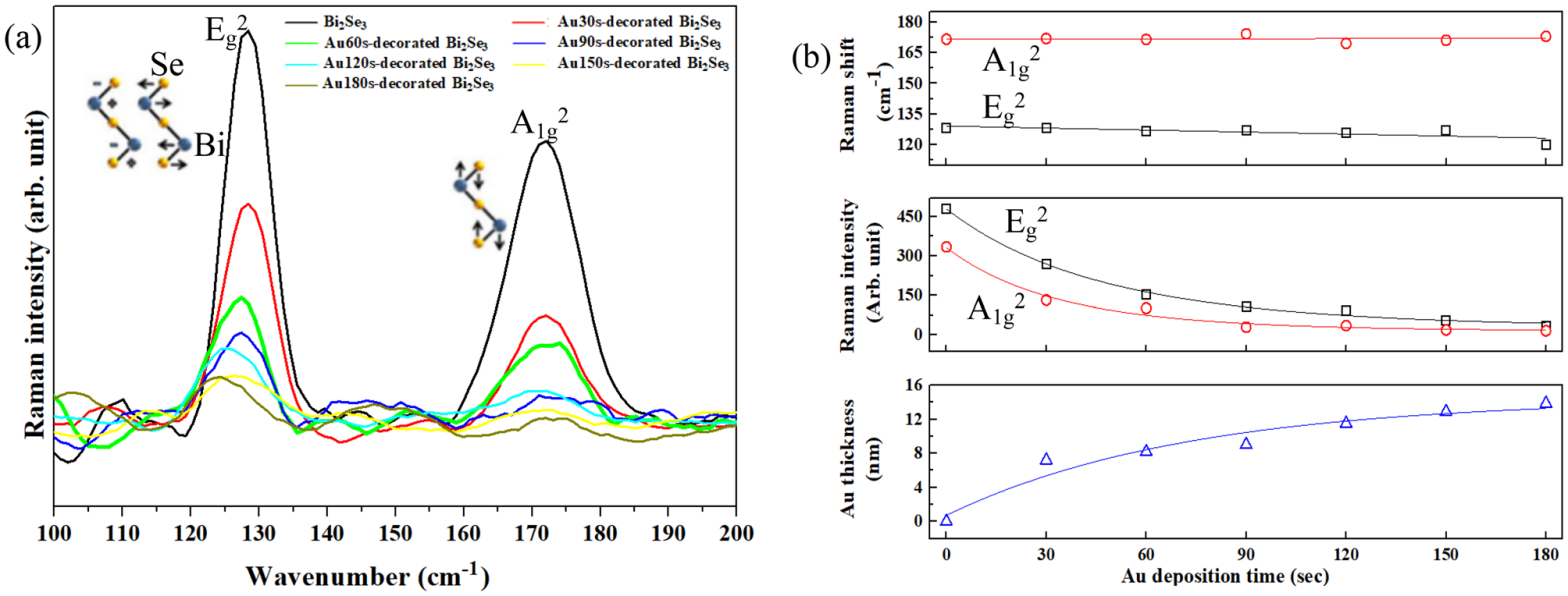
(**a**) Raman spectra of the pristine Bi_2_Se_3_ and Au-decorated Bi_2_Se_3_ NPs. (**b**) Raman shifts and intensities of E_g_^2^ and A_1g_^2^ mode, and Au thickness with various Au deposition time.

Figure 5a presents typical Raman shifts of Bi_2_Se_3_ in the modes of E_g_^2^ and A_1g_^2^. Bi_2_Se_3_ has a layered structure. Each layer comprises five atomic layers, Se^1^–Bi–Se^2^–Bi–Se^1^, and is called a quintuple layer (QL). Covalent bonds dominate bonding within each QL; the van der Waals’ forces bond QLs^59,60^. Two significant vibration modes are observed at around 125 cm^−1^ and 170 cm^−1^, which are denoted as E_g_^2^ and A_1g_^2^. E_g_^2^ is the symmetric in-plane bending mode, involving shearing of the upper two layers of Se^1^–Bi atoms, which vibrate in opposite directions, as shown in Fig. 5a. A_1g_^2^ is the symmetric out-of-plane stretching mode that is associated with Se^1^–Bi atoms that do stretch in opposite directions^11^.

Figure 5b presents the positions and intensities of the A_1g_^2^ and E_g_^2^ modes, and Au thickness of Au-decorated Bi_2_Se_3_ NPs. The position of the A_1g_^2^ modes remain constant as Au thickness increases, while those of E_g_^2^ modes are red-shifted. The following several factors affect the position of the A_1g_^2^ and E_g_^2^ modes. (1) Calcination temperature, as the formation of the oxygen vacancies causes a blue-shift of the mode position^61^. (2) The metal nanoparticles that decorate on the metal oxide surface red-shift the E_g_ Raman mode^62^. (3) Tensile and compressive stresses produce red- and blue-shift of the Raman vibration modes, respectively^63,64^. (4) Weakened bonding causes red Raman shifts^63^. On account of the fabrication process in this work and the results provided above, the red-shift of the E_g_^2^ mode in this work is attributed to the decoration by Au particles of the surface of the Bi_2_Se_3_ NPs. Additionally, increasing the Au thickness did not affect the A_1g_^2^ Raman position.

The Raman intensities, shown in Fig. 5b, in modes A_1g_^2^ and E_g_^2^ decrease as the Au thickness increases. The following factors affect the Raman intensity. (1) Lattice distortion of the host material, related to doping and the formation of the oxygen vacancies, reduces Raman intensity owing to the re-symmetrized crystal structure^65^. (2) A structural phase transition changes the lattice constants and thereby the Raman intensity^66^. (3) The temperature of the measuring system causes a phase transition and changes the Raman intensity^65^. (4) The covering of the sample surface with a layer reduces the Raman intensity^65^. (5) Increasing the thickness of the NPs increases the Raman intensity^63^. (6) The screening effect of the metal layers reduces the Raman intensity^63^. Based on the above, the decreases of the Raman intensities in modes A_1g_^2^ and E_g_^2^ are attributed to factors 4 and 6. The Raman results in Fig. 5a, b suggest that the Au thickness affects the intensities in different Raman modes more than it affects the position of the Raman peaks.

### Analysis of UV–visible light optical property and the proposed mechanism

**Figure 6:**
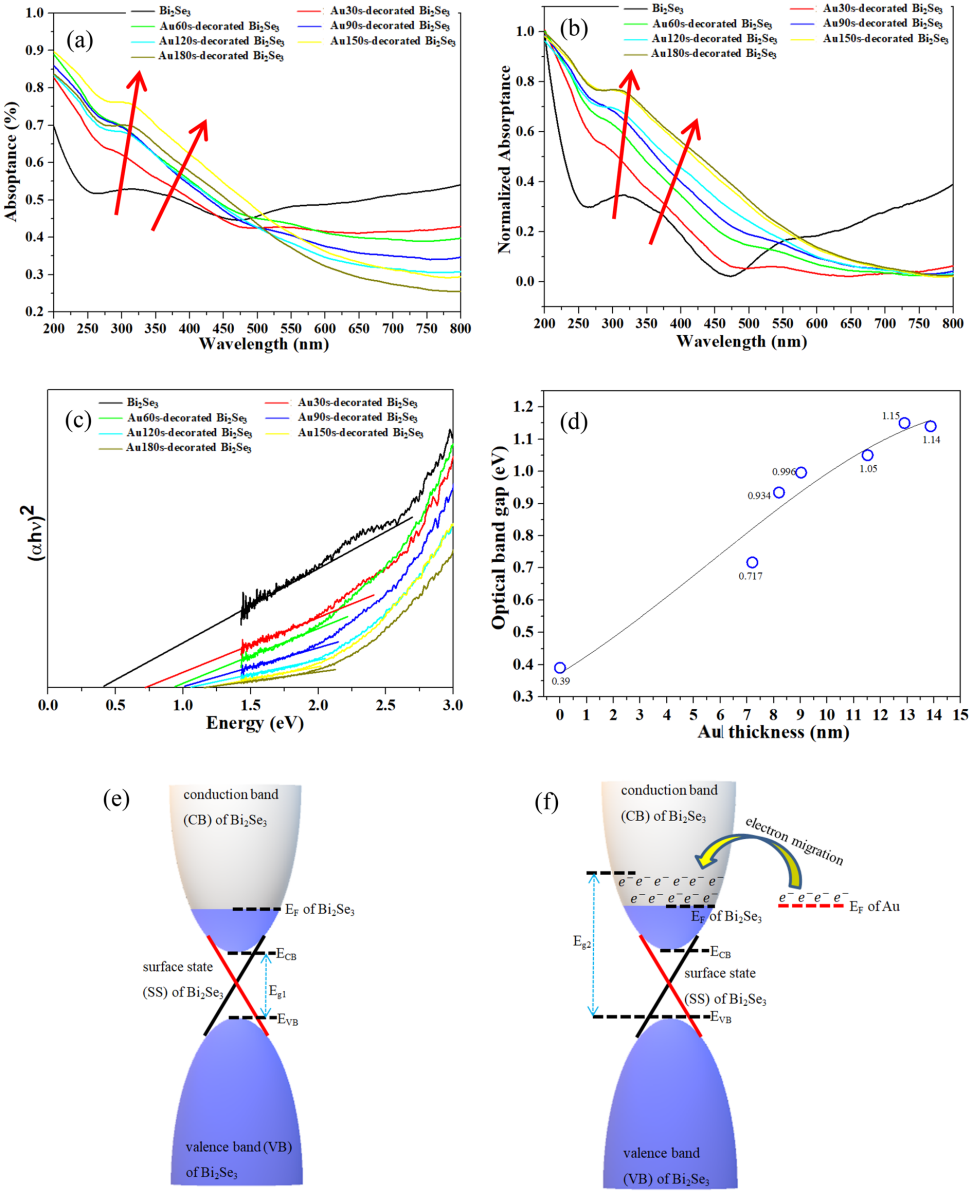
UV–visible spectra of (**a**) absorptance, (**b**) normalized absorptance, (**c**) Tauc-plot, and (**d**) optical band gap of Au-decorated Bi_2_Se_3_ NPs. (**e**) the schematic band structures of the pure Bi_2_Se_3_, and (**f**) the proposed mechanism of the electron migration between the Bi_2_Se_3_ and Au.

Figure 6a presents the absorptance of Au-decorated Bi_2_Se_3_ NPs. It reveals that absorptance increases Au thickness at wavelengths under around 500 nm; it decreases at wavelengths above than 500 nm. The absorptance spectra are simulated using the software TFCale, as shown in Fig. S3. Figure S3a presents a simulated absorptance spectrum of an Au thin film/sapphire-substrate. The thicknesses of the examined Au thin films are 0, 3, 6, 9, 12, 15, 18, and 20 nm. This simulation shows that increasing the Au thickness increases the absorptance in the range from 200 to 800 nm. Figure S3b plots the absorptance of the Au thin film/Bi_2_Se_3_ thin film/sapphire-substrate, and the results are the experimental results that were shown in Fig. 6a. The thickness of the Bi_2_Se_3_ thin film is 73 nm, and the Au thicknesses are 0, 3, 6, 9, 12, 15, 18, and 20 nm. The simulated spectra exhibit a similar tendency as the experimental spectra, supporting this observation. The normalized absorptance spectra, shown in Fig. 6b, reveal a significant red-shift of the absorptance peak at around 300 nm and that same shifts from 375 to 325 nm and 400 nm as in Fig. 6a. This phenomenon is attributed to the increased Au thickness on the Bi_2_Se_3_ NPs^30^. These results reveal that the absorptance is significantly affected by the thickness of the Au, and proves that the incident energy of the 488 nm laser decreases as the Au thickness increases; therefore, the Raman intensities of Au-decorated Bi_2_Se_3_ NPs (Fig. 5a) decrease as the Au thickness increases.

The optical band gap of Au-decorated Bi_2_Se_3_ NPs is estimated from a Tauc plot, as follows^67^.

6 $$\left( {\alpha h\nu } \right)^{n} = A\left( {h\nu - E_{g} } \right)$$

where *α* is the absorption coefficient; *h* is Planck’s constant; *ν* is the frequency of the incident light; the characteristic coefficient of n for Bi_2_Se_3_ is 2; *A* is a constant; *E*_*g*_ is the optical band gap energy. Figure 6c,d show Tauc-plots and the estimated optical band gaps of the Au-decorated Bi_2_Se_3_ NPs, respectively. The pristine Bi_2_Se_3_ NPs have a band gap of 0.39 eV similar to that obtained in previous report^68^, whereas the optical band gaps of the Au-decorated Bi_2_Se_3_ NPs linearly increase with the Au thickness, as plotted in Fig. 6d.

These results show that the Au thickness significantly affects the optical properties of the Au-decorated Bi_2_Se_3_ NPs, including absorptance and the optical band gap. The concentration of the free electrons applied by the Au nanoparticles is enhanced with increasing the amounts of the Au nanoparticles, i.e., the thicker Au layer. The concentration of the electrons that migrate to the surface of the Bi_2_Se_3_ NPs should be enhanced as well. Hence, the free electrons in Au migrate to the conduction band (CB) of the Bi_2_Se_3_, and then the CB bottom of the Bi_2_Se_3_ is occupied. The excitation, therefore, of the electrons in the valence band of the Bi_2_Se_3_ need more energy to transit to the CB. This phenomenon is attributed to the Burstein-Moss effect^69^, and thus the optical band gap of the Au-decorated Bi_2_Se_3_ NPs increases. Therefore, the energy gap of E_g1_ in the pure Bi_2_Se_3_ NPs is smaller than that of the E_g2_ in the Au-decorated Bi_2_Se_3_ NPs. Figure 6e,f present the schematic band structures of the pure Bi_2_Se_3_ and the proposed mechanism between the Bi_2_Se_3_ and Au, respectively.

## Conclusions

The thickness of the Au significantly affects the optical band gaps and absorptance of Au-decorated Bi_2_Se_3_ NPs. The optical band gap linearly increases with Au thickness. Pristine Bi_2_Se_3_ NPs have a band gap of 0.39 eV. Au-decorated Bi_2_Se_3_ NPs with the thicker Au (13.88 nm) have a band gap of 1.14 eV, which is 1.92-fold larger than that of the pristine Bi_2_Se_3_. The increased optical band gap of the Au-decorated Bi_2_Se_3_ NPs is attributable to the Burstein-Moss effect. A thicker Au provides greater absorptance of Au-decorated Bi_2_Se_3_ NPs in the wavelength range from 200 and 500 nm, but lower absorptance between 500 to 800 nm. Both of these findings are confirmed by optical simulation using the software TFCale. The formation of rhombohedral Bi_2_Se_3_ and the FCC Au phase are confirmed by XRD and HRTEM, which reveal that Au has no effect on the Bi_2_Se_3_ crystal structure. Raman spectra show a red-shift of the E_g_^2^ mode due to the decoration by Au particles of the surface of the Bi_2_Se_3_ NPs, indicating that Au bonds with the Se atoms at the surface between Au and Bi_2_Se_3_ NPs. XPS results confirm the formation of Bi-Se bonds in the Bi_2_Se_3_ phase and a charge transition between Au and Bi_2_Se_3_, which are revealed by a shift in binding energy. Owing to the screening effect of the Au, the peak intensities in the XPS and Raman spectra decrease as the Au thickness increases. This work reveals that the thickness of the Au affects the optical band gaps and absorptance of the Au-decorated Bi_2_Se_3_ NPs. The increased absorptance in the shorter wavelength region (λ < 500 nm) and the increased transmittance in the longer wavelength region (λ > 500 nm) favor the use of Au-decorated Bi_2_Se_3_ NPs.

## Methods

### Synthesis of Bi_2_Se_3_ and Au-decorated Bi_2_Se_3_ nanoplatelets

A catalyst-free vapor–solid mechanism is used to fabricate pristine Bi_2_Se_3_ nanoplatelets (NPs) on an Al_2_O_3_(100) substrate (0.5 × 0.5 mm^2^) by thermal chemical evaporation deposition in a quartz tube furnace. Mixed metallic powders of 0.1 g bismuth (purity = 99%, 4.78 × 10^−4^ mol, Merck, Darmstadt, Germany) and 0.1 g selenium (purity = 99%, 1.27 × 10^−3^ mol, Alfa Aesar, Ward Hill, MA, USA) were used as precursors and placed in an alumina boat, which was put in the central heating zone of a quartz tube, which was heated to the setting point of 600 °C at a rate of 10 °C/min under a pressure of 1.0 × 10^−2^ Torr; these conditions were maintained for 60 min. The substrates were placed upstream in the quartz tube at 140 °C, 21 cm away from the alumina boat. Pristine Bi_2_Se_3_ NPs were subsequently grown on the Al_2_O_3_(100) substrate. After 60 min of synthesis, the system was cooled to room-temperature. An Au thin film was deposited on a sapphire substrate for 150 s (110 V, 5 mA) at 7.5 × 10^−2^ Torr, and then a profilometer (Dektak XT, Bruker, Billerica, MA, USA) was used to estimate the rate of deposition of Au thin film. Au was deposited on the Bi_2_Se_3_ NPs at a working distance of 20 mm by magnetron sputtering (110 V, 5 mA) using a 2-inch Au target at room-temperature under a working pressure of 7.5 × 10^−2^ Torr. Au was deposited for 30, 60, 90, 120, 150, and 180 s, yielding Au30s-, Au60s-, Au90s-, Au120s-, Au150s- and Au180s-decorated Bi_2_Se_3_ NPs, respectively.

### Characterization of Au-decorated Bi_2_Se_3_ nanostructures

The crystal structures of the pristine Bi_2_Se_3_ and Au-decorated Bi_2_Se_3_ NPs were characterized by a grazing incidence small-angle X-ray diffractometer (λ = 0.154 nm, 30 A, 40 kV, Bruker MXP-III ) at 2θ = 15°–50°, and a high-resolution electron transmission microscope (HRTEM, JEOL JEM-2010, Tokyo, Japan). X-ray photoelectron spectroscopy (XPS, ULVAC-PHI PHI 5000 VersaProbe/Scanning Electron Spectroscopy for Chemical Analysis microscope, Tokyo, Japan) with an X-ray source of Al kα (1486.6 eV) and Raman spectroscopy (3D Nanometer-Scale Raman PL Microspectrometer, Tokyo Instruments, INC., Tokyo, Japan) with a semiconductor laser (λ = 488 nm) were used to determine the bonding energies and vibration modes of the Au-decorated Bi_2_Se_3_ NPs. The surface morphology of the Au-decorated Bi_2_Se_3_ NPs was observed using high resolution Field Emission Scanning Electron Microscopy (ZEISS Ultra Plus, Carl Zeiss Microscopy GmbH, Oberkochen, Germany). The optical absorptance from λ = 200 to 800 nm was measured using a UV–visible spectrometer (Hitachi U3900-H, Hitachi Ltd., Tokyo, Japan) with an integrating sphere.

## Supplementary Information

Supplementary Figures.

## Data Availability

The datasets used and/or analysed during the current study available from the corresponding author on reasonable request.
