# Supplementary material for: Fabrication, characterization and optical properties of Au-decorated Bi2Se3 nanoplatelets
Source: Sci Rep. 2022 Oct 22;12:17761. doi: 10.1038/s41598-022-22408-5 (PMC9587984; doi:10.1038/s41598-022-22408-5)
Supplement: Supplementary file 1 — Supplementary Figures. [file 41598_2022_22408_MOESM1_ESM.docx]

|   (a)  (c) |   (b)  (d) |
| --- | --- |
|   (e) |   (f) |
|   (g) |  |
|  |  |
| **Figure S1.** FESEM images of (a) pristine Bi_2_Se_3_, (b) Au30s-, (c) Au60s-, (d) Au90s-,  (e) Au120s-, (f) Au150s-, and (g) Au180s-decorated Bi_2_Se_3_ nanoplatelets. | |

| 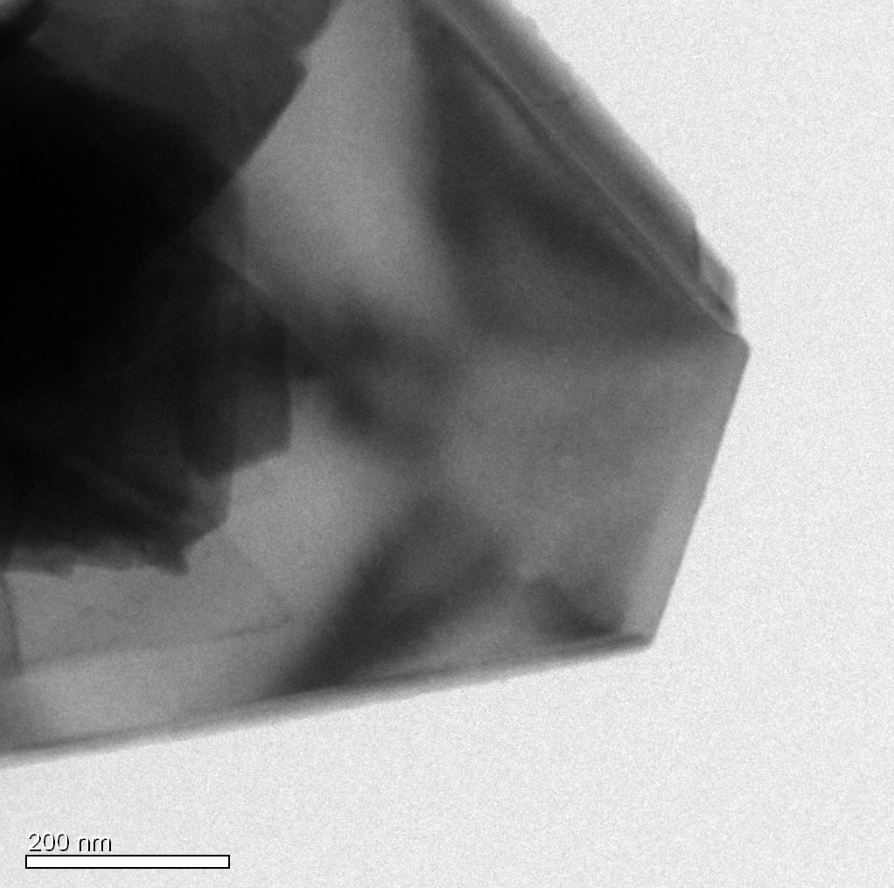 | 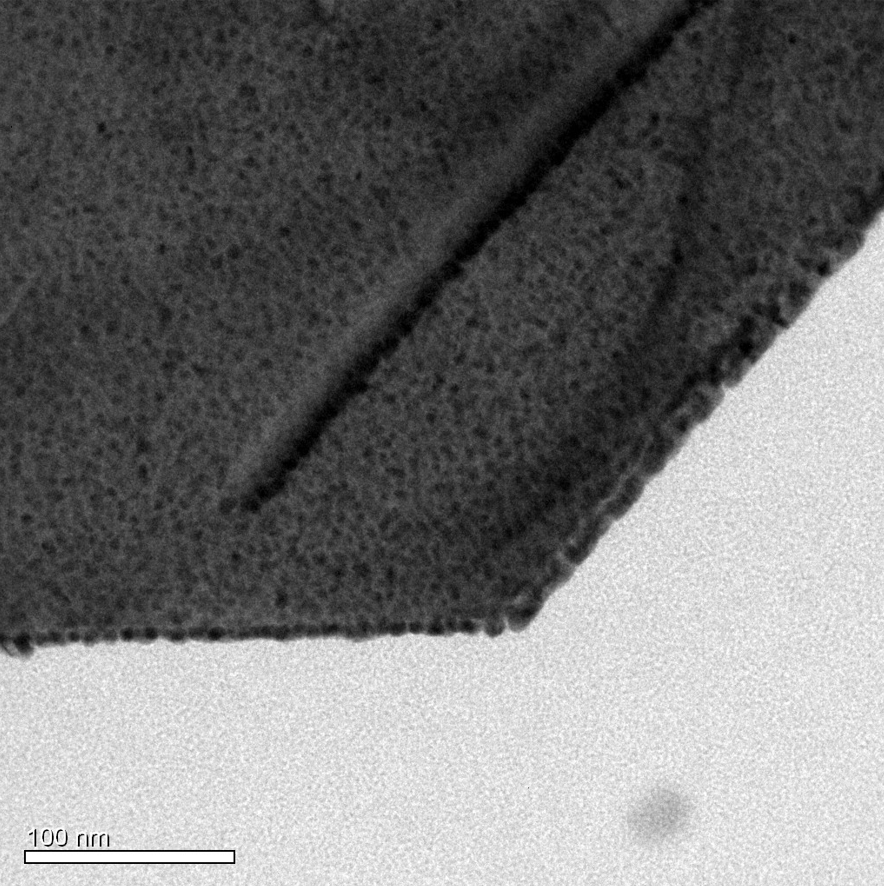  **7.2 nm**  (b)  (d)  (g)  (e)  (a)  (c) |
| --- | --- |
| 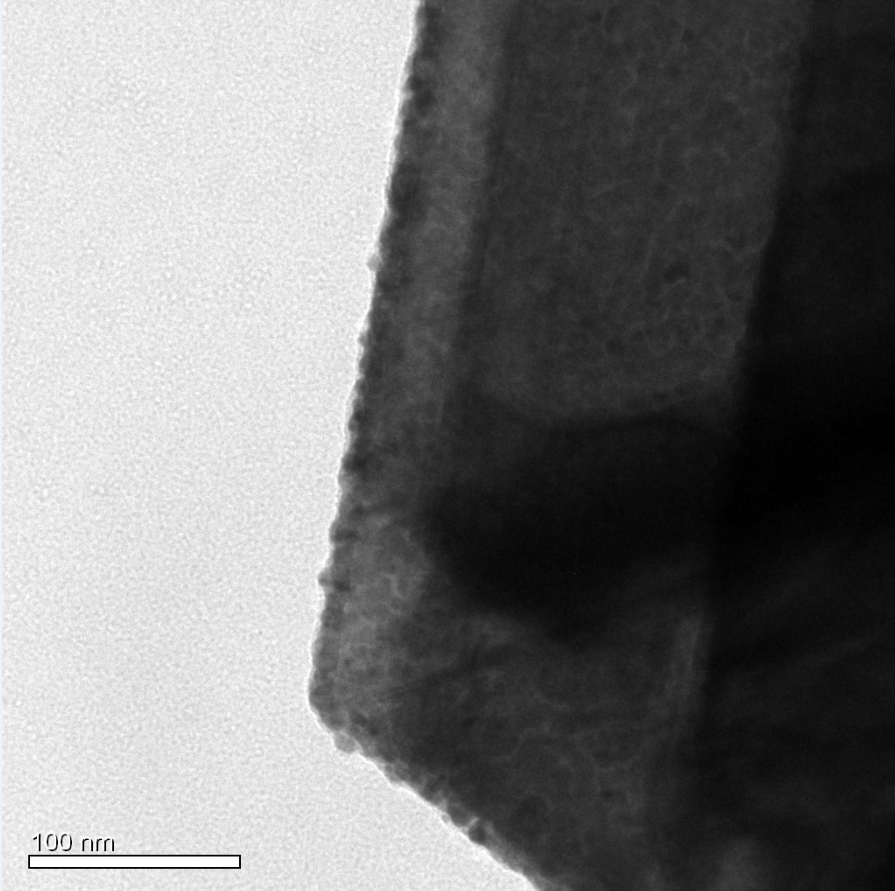  **8.2 nm** | 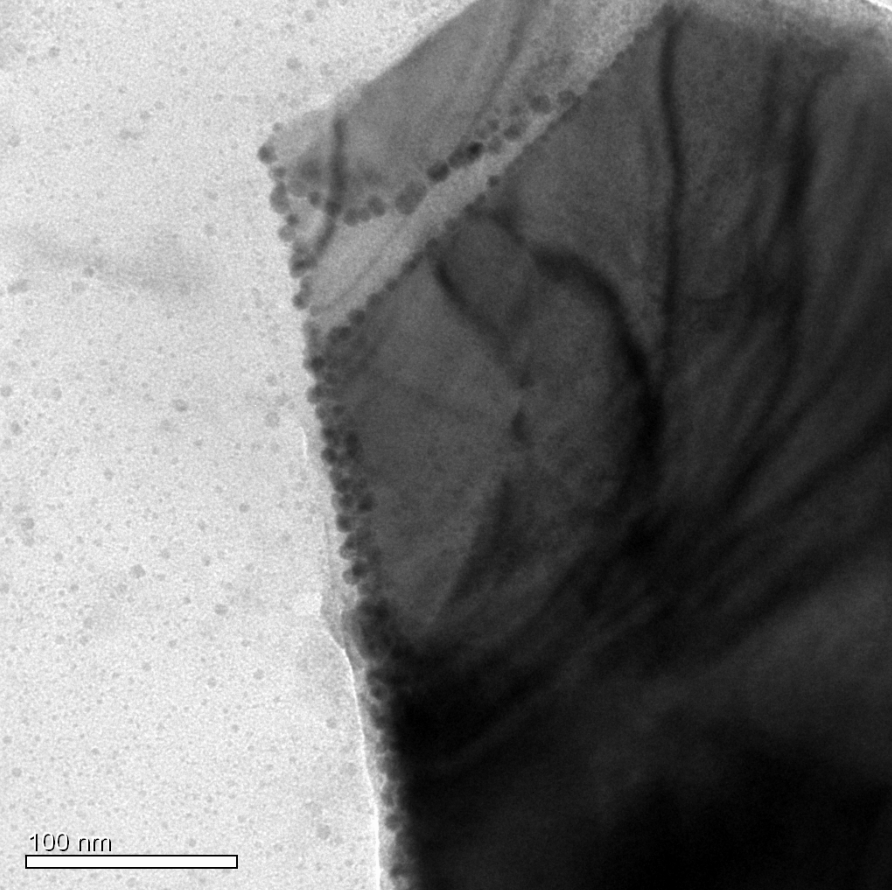  **9.03 nm**  (f) |
| 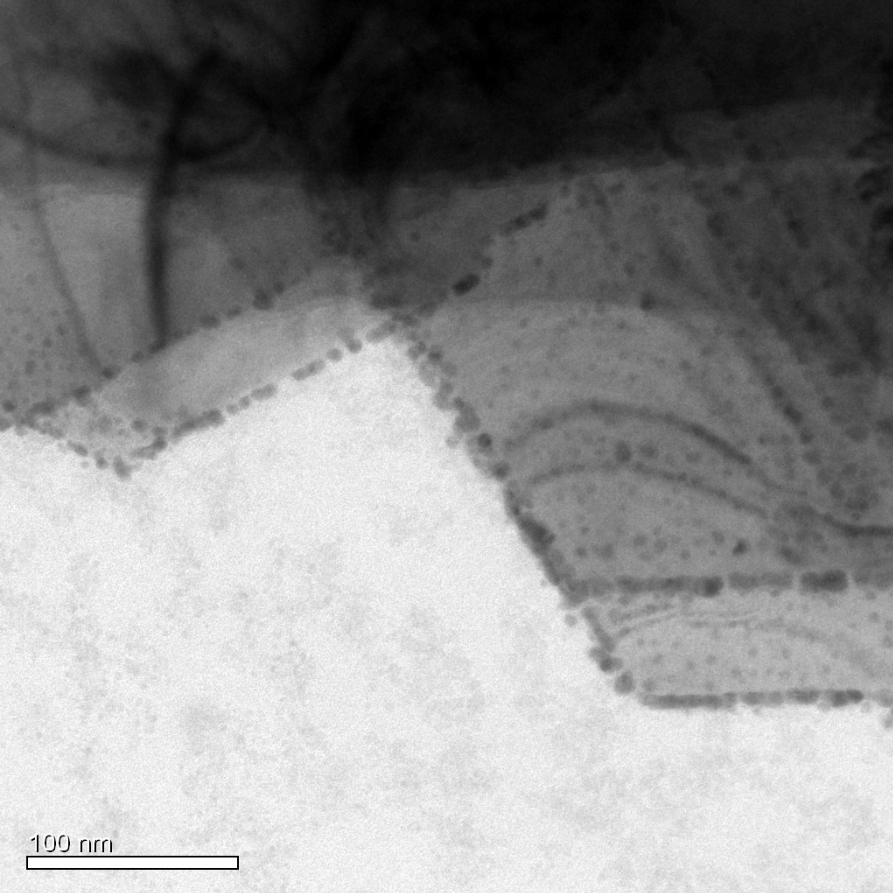  **11.52 nm** | 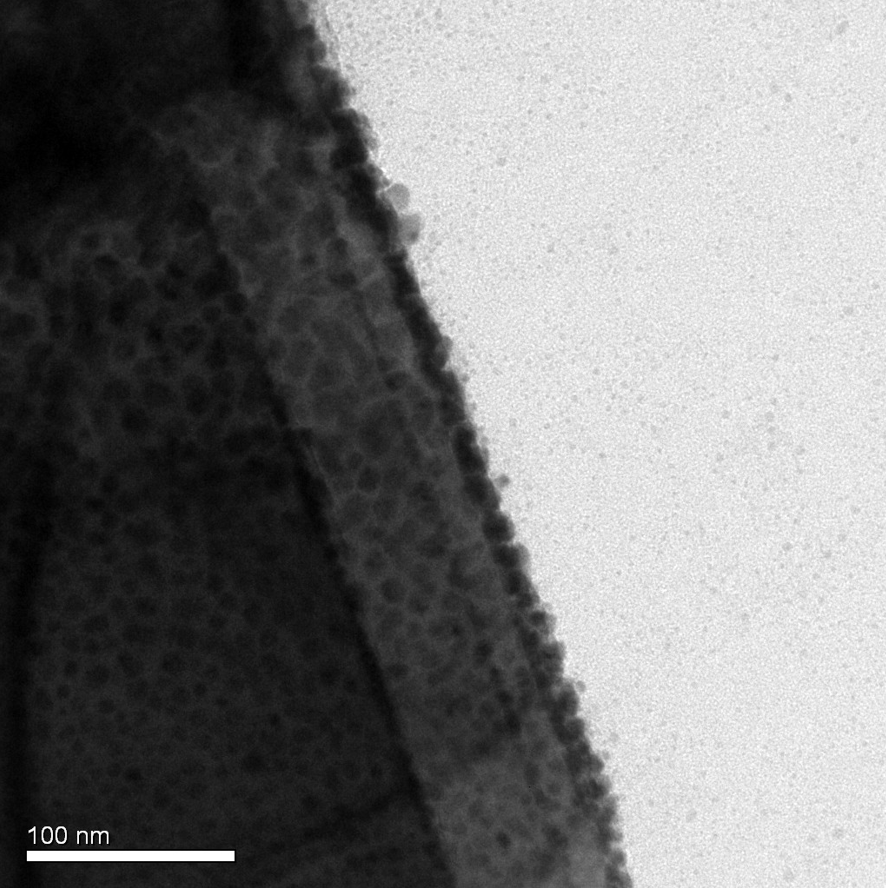  **12.9 nm** |
| 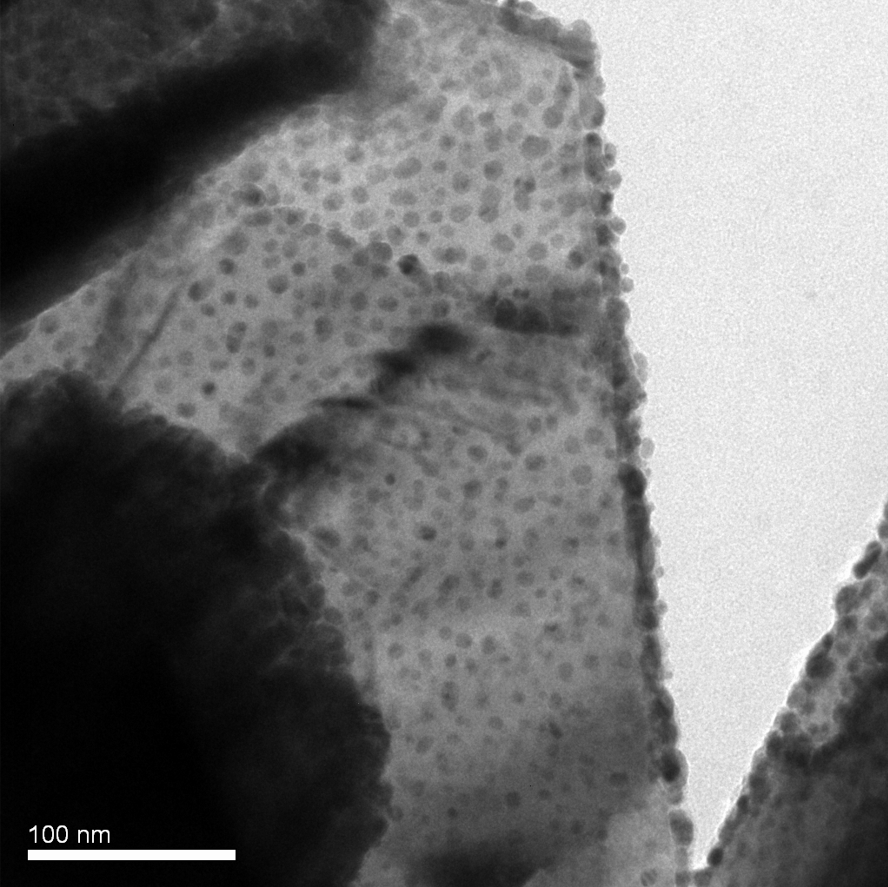  **13.88 nm** |  |
| **Figure S2.** HRTEM images of (a) pristine Bi_2_Se_3_, (b) Au30s-, (c) Au60s-, (d) Au90s-, (e) Au120s-, (f) Au150s-, and (g) Au180s-decorated Bi_2_Se_3_ nanoplatelets. | |

| 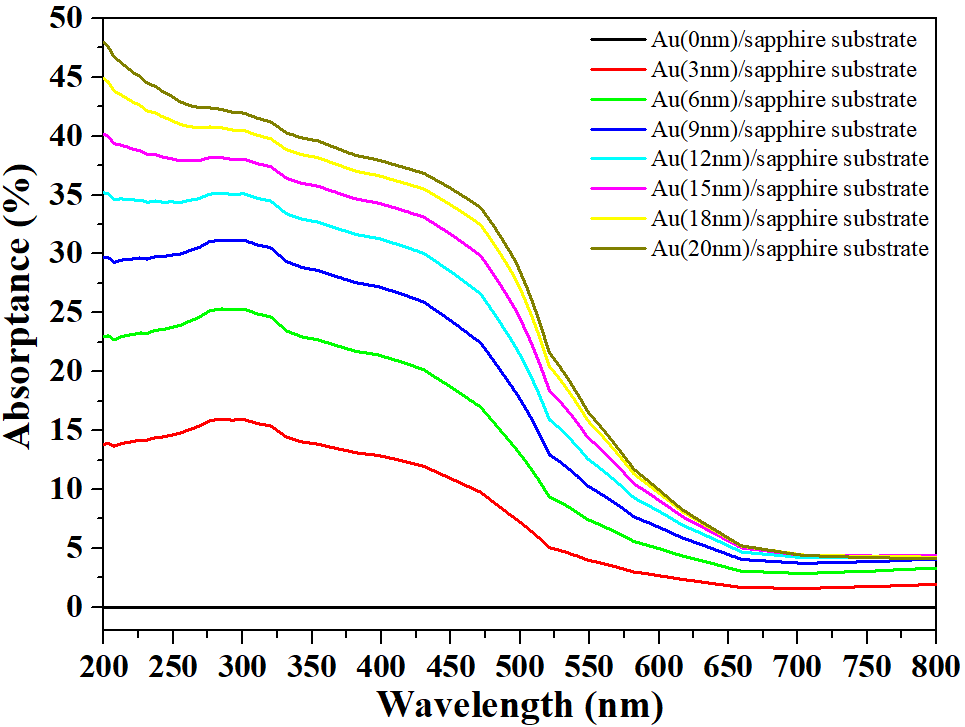  (b)  (a) | 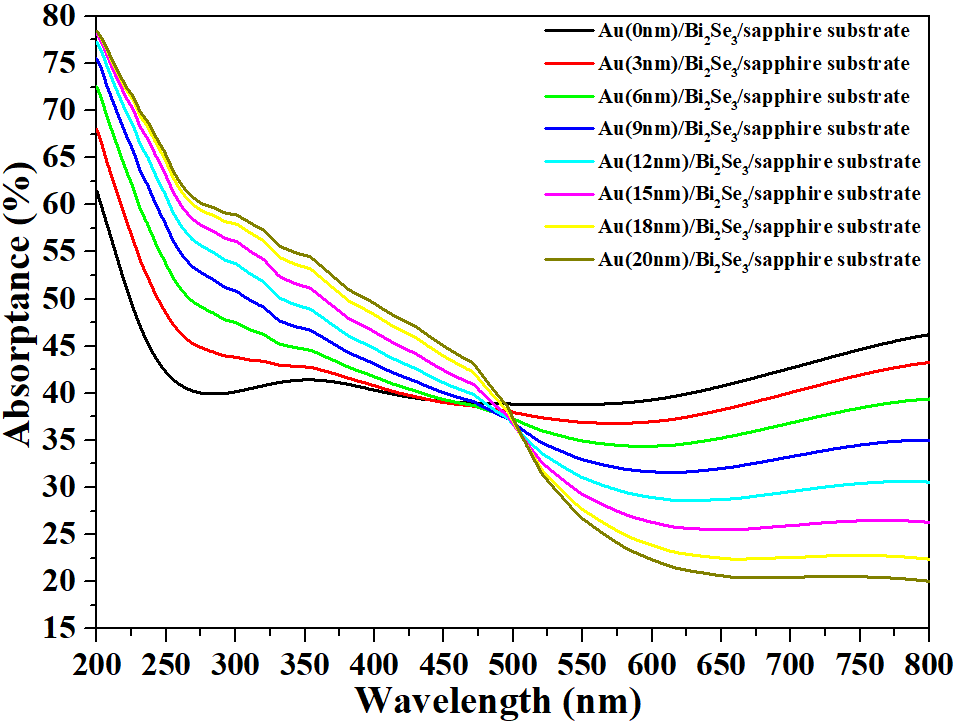 |
| --- | --- |
| **Figure S3.** TFCale simulation results of (a) Au thin film/sapphire substrate and (b) Au thin film/Bi_2_Se_3_ thin film/sapphire substrate. | |
